# Sequential organogenesis sets two parallel sensory lines in medaka

**DOI:** 10.1242/dev.142752

**Published:** 2017-02-15

**Authors:** Ali Seleit, Isabel Krämer, Elizabeth Ambrosio, Nicolas Dross, Ulrike Engel, Lázaro Centanin

**Affiliations:** 1 Animal Physiology and Development, Centre for Organismal Studies (COS) Heidelberg, Im Neuenheimer Feld 230, Heidelberg 69120, Germany; 2 The Hartmut Hoffmann-Berling International Graduate School of Molecular and Cellular Biology (HBIGS), University of Heidelberg, Heidelberg, Germany; 3 Nikon Imaging Center at the University of Heidelberg, Heidelberg, Germany

**Keywords:** Organogenesis, Lateral line, Cxcr4b, Cxcr7, Eya1, Neuromast

## Abstract

Animal organs are typically formed during embryogenesis by following one specific developmental programme. Here, we report that neuromast organs are generated by two distinct and sequential programmes that result in parallel sensory lines in medaka embryos. A ventral posterior lateral line (pLL) is composed of neuromasts deposited by collectively migrating cells whereas a midline pLL is formed by individually migrating cells. Despite the variable number of neuromasts among embryos, the sequential programmes that we describe here fix an invariable ratio between ventral and midline neuromasts. Mechanistically, we show that the formation of both types of neuromasts depends on the chemokine receptor genes *cxcr4b* and *cxcr7b*, illustrating how common molecules can mediate different morphogenetic processes. Altogether, we reveal a self-organising feature of the lateral line system that ensures a proper distribution of sensory organs along the body axis.

## Abstract (precis)

**Summary:** Two parallel sensory lines in medaka share a common origin and are composed of identical organs that are, nevertheless, generated through different morphogenetic programmes.

## INTRODUCTION

The diversity of body forms across the animal kingdom is astonishing. Even within taxa, species show major differences in body size and relative shape. Teleost fish are arguably the most extreme example of such diversity among vertebrates, considering the range from a seahorse to an eel, a pufferfish, a tuna and a zebrafish. When reviewing all these diverse forms, we wondered how peripheral sensory systems could adapt to such dissimilar body shapes. Here, we tackle this question by focusing on the embryonic formation of the posterior lateral line (pLL) system in medaka (*Oryzias latipes*).

The lateral line system is composed of neuromasts, differentiated organs containing dozens of cells that sense the direction of water flow and relay this information to the brain (Ghysen and Dambly-Chaudière, 2007). The arrangement of neuromasts on the head, main body and tail is species specific (Sapède et al., 2002) and relates to the body shape of the fish (Coombs et al., 2014, 1988). Most of our knowledge on lateral line development comes from studies on the pLL of zebrafish; several research groups have contributed molecular, genetic and physiological approaches that have deepened our understanding on the morphogenesis, homeostasis and regeneration of the system (Aman and Piotrowski, 2008; David et al., 2002; Grant et al., 2005; Haas and Gilmour, 2006; Hernández et al., 2006; Lecaudey et al., 2008; López-Schier and Hudspeth, 2005, 2006; Lush and Piotrowski, 2014; Ma and Raible, 2009; McGraw et al., 2014; Nechiporuk and Raible, 2008; Pichon and Ghysen, 2004; Sapède et al., 2002; Sánchez et al., 2016). The formation of the pLL starts with a group of cells (a primordium) that delaminates near the otic vesicle from the lateral line placode and migrates posteriorly along the horizontal myoseptum (Ghysen and Dambly-Chaudière, 2007; Kimmel et al., 1995). A first primordium (primI) deposits five to eight pre-formed neuromasts from its rear edge (Ghysen and Dambly-Chaudière, 2007; Lecaudey et al., 2008; Nechiporuk and Raible, 2008) at regular intervals, which are connected by so-called inter-neuromast cells (ICs). This is followed by a second primordium (primII) that runs along the same path to deposit additional neuromasts between the ones previously generated by primI (Ghysen and Dambly-Chaudière, 2007; Ledent, 2002; Sapède et al., 2002). Both sets of neuromasts migrate ventrally (Ghysen and Dambly-Chaudière, 2007; Ledent, 2002; Whitfield, 2005), and during the first post-embryonic days new neuromasts are formed by proliferation of the ICs (Grant et al., 2005; López-Schier and Hudspeth, 2005; Lush and Piotrowski, 2014; Whitfield, 2005) to complete a pLL system that displays roughly one neuromast per somite boundary. There are additional primordia that generate the dorsal pLLs (Ghysen and Dambly-Chaudière, 2007; Nuñez et al., 2009; Sapède et al., 2002), and these and other observations have contributed to the notion that the different pLLs in fish are each generated by different primordia.

The anterior-to-posterior migration of primI depends on Cxcl12a/Cxcr4b/Cxcr7b signalling (David et al., 2002; Haas and Gilmour, 2006; Valentin et al., 2007); the migrating primordium has been shown to generate its own Cxcl12a gradient via the opposing roles of the two chemokine receptors Cxcr4b and Cxcr7b (Donà et al., 2013; Venkiteswaran et al., 2013). In fact, primI fails to migrate in mutants for *cxcr4b*, *cxcr7b* or *cxcl12a* in zebrafish. In a series of elegant transplantation experiments, it was shown that a few wild-type cells can rescue the migration of a *cxcr4b* mutant primI (Haas and Gilmour, 2006). Interestingly, rescue by wild-type cells occurs if these are located at the leading edge of the primordium, which nevertheless deposits mutant cells at the rear to generate all the neuromasts of the pLL system (Haas and Gilmour, 2006). Therefore, primordium migration can be achieved with just a few cells that receive and process the ligand information.

Our current understanding of how the pLL develops in other fish is limited due to the lack of proper genetic tools to characterise the morphogenetic process dynamically. In the context of the diversity of body shapes among teleosts, the ‘one-primordium one-lateral-line’ concept would imply the existence of more primordia in fish displaying more lateral lines. Interestingly, medaka embryos are larger than zebrafish embryos and display an extra lateral line at the time of hatching. We therefore decided to expand the analysis of the pLL system to medaka, which offers extensive transgenic and experimental embryology approaches (Kirchmaier et al., 2015), to address how diverse distributions of sensory organs arise during development.

We generated a transgenic line in medaka that specifically labels neuromasts using the *eyes-absent-1* (*eya1*) promoter to follow the dynamic formation of the pLL *in vivo*. We observed that neuromasts of two parallel pLLs in medaka are formed in a sequential manner by cells coming from a common primordium. The first set of organs are deposited from the rear of a migrating primordium and shortly after start to migrate ventrally forming the ventral pLL. A second set of neuromasts is formed by ICs and cells that escape from previously formed organs, organise into a cluster and migrate dorsally to form the midline pLL. We demonstrate that Cxcr4b and Cxcr7 are used iteratively: initially to drive the migration of the primordium and afterwards in individual cell migration during secondary organ formation. Finally, we show that this mode of organogenesis, whereby already generated neuromasts can produce second generation organs, is controlled within the lateral line and propose that this could facilitate the adaptation of the sensory system to the variety of body shapes found among teleost fish.

## RESULTS

### Alternated organ distribution between two parallel pLLs

**Fig. 1.**
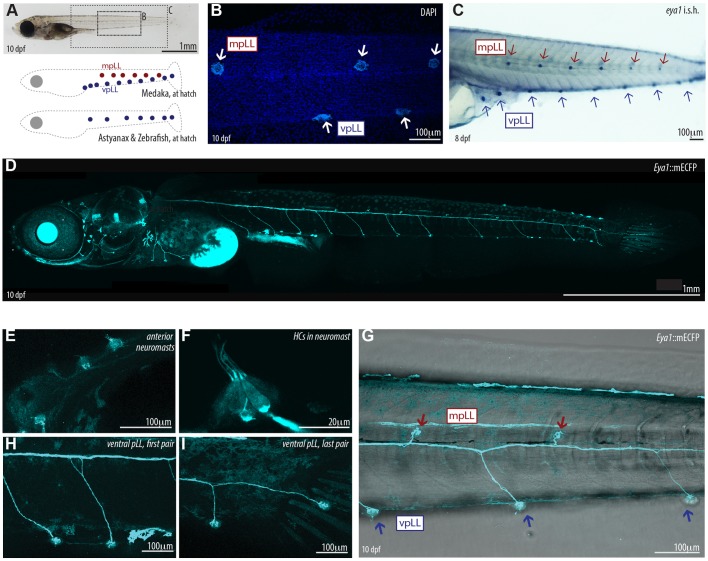
**The *Eya1*:mCFP transgenic line labels neuromasts in medaka.** (A) Whereas *Astyanax* and zebrafish have a single ventral pLL at the end of embryonic development, medaka shows an additional line at the horizontal myoseptum – the mpLL. (B) Neuromasts are easily identified by DAPI staining on fixed embryos. (C) *In situ* hybridisation shows strong expression in mpLL and vpLL neuromasts in a stage 39 medaka embryo. (D) Tg(*Eya1*:mECFP) labels neuromasts and their neural connections *in vivo*. Composite of seven individual frames stitched together using a Fiji macro. (E,F) Detail of neuromasts in the anterior lateral line (E), and hair cells (HCs) (F). (G-I) Alternating distribution of midline and ventral pLL neuromasts (G) along the trunk of a medaka larva, with exceptions of the first two pairs (H) and the last pair (I) of ventral pLL neuromasts.

At the end of embryogenesis, the pLL system of medaka exhibits two parallel sets of mature neuromasts, arranged ventrally and on the midline (Fig. 1A-C) (Ishikawa, 1994; Sapède et al., 2002; Wada et al., 2008; Yasuoka et al., 2004). This differs from the embryonic patterns of other teleost fish such as zebrafish or *Astyanax*, in which most neuromasts are found along one line (Fig. 1A) (Sapède et al., 2002). The additional pLL in medaka is believed to originate from an independent primordium, a speculation that has not yet been formally tested. When quantifying the neuromasts in each pLL, we noticed that ventral and midline neuromasts are arranged in staggered rows, where each neuromast in the midline pLL (mpLL) is surrounded by an anterior and a posterior neuromast in the ventral pLL (vpLL) (99.2%; *n*=141 neuromasts, *n*=22 pLLs) (Fig. 1B,C). The two-to-three most anterior and the two-to-three most posterior neuromasts of the ventral pLL do not have a counterpart in the midline (22/22 pLL and 21/22 pLL, respectively) (Fig. 1C). The alternated pattern of neuromasts in the ventral and midline pLL suggests a coordinated, rather than a complementary, organogenesis between the two pLLs.

To follow lateral line formation in medaka *in vivo*, we created transgenic lines in which the promoter of the medaka *eyes-absent 1* gene (*eya1*) drives the expression of different fluorescent proteins. Previous studies indicated that *eya1* is a bona fide marker for the lateral line in medaka, as *eya1* mRNA *in situ* hybridisation labels neuromasts during embryonic and early post-embryonic stages (Fig. 1C) (Wada et al., 2008). Our transgenic lines recapitulate medaka *eya1* expression (Fig. 1D) and allow *in vivo* visualisation of neuromasts during embryonic, juvenile and adult stages (Fig. 1D-I; Fig. S1). The alternated distribution of neuromasts in Tg(*Eya1*:EGFP) embryos was identical to non-transgenic controls (99%; *n*=197 midline neuromasts, *n*=32 pLLs), with around ten neuromasts in the ventral pLL (10.1±0.9; *n*=126 pLLs) and between six and seven in the midline pLL (6.6±0.8; *n*=126 pLLs). All mature neuromasts from the different post-embryonic lateral lines were labelled by Tg(*Eya1*:EGFP) and by Tg(*Eya1*:H2B-EGFP) as indicated by co-staining with specific vital dyes (DiAsp, DAPI; Fig S1) for the differentiated neurons of the organ (*n*>100 neuromasts, *n*>10 larvae for each transgenic line). In addition, our transgenic lines allow the visualisation of immature neuromasts that have not yet generated differentiated cells (Fig. S1). Therefore, Tg(*Eya1*:EGFP) recapitulates the endogenous *eya1* expression and allows neuromast formation to be visualised *in vivo*.

### Sequential formation of vpLL and mpLL neuromasts from a common primordium

**Fig. 2.**
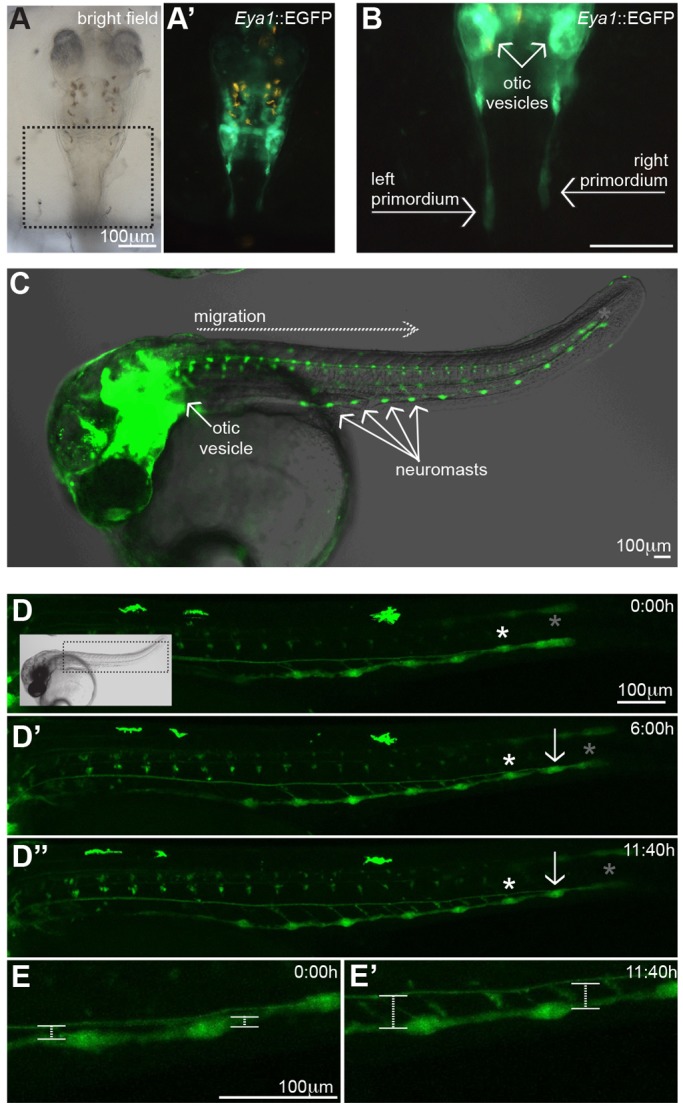
**Primordium migration during pLL formation in medaka.** (A-B) A primordium detaches near the otic vesicle at stage 26 and starts migrating posteriorly. B shows a detailed view of the boxed area in A. (C-E) The primordium (grey asterisks) migrates through the horizontal midline and deposits neuromasts (white asterisks) at regular intervals (C-D″). Soon after deposition, neuromasts start migrating ventrally (D′-E′) while the primordium continues towards the caudal fin and keeps depositing new organs along the horizontal myoseptum (arrow in D-D″″). Boxed area in D indicates the field of view in D-D″.

**Fig. 3.**
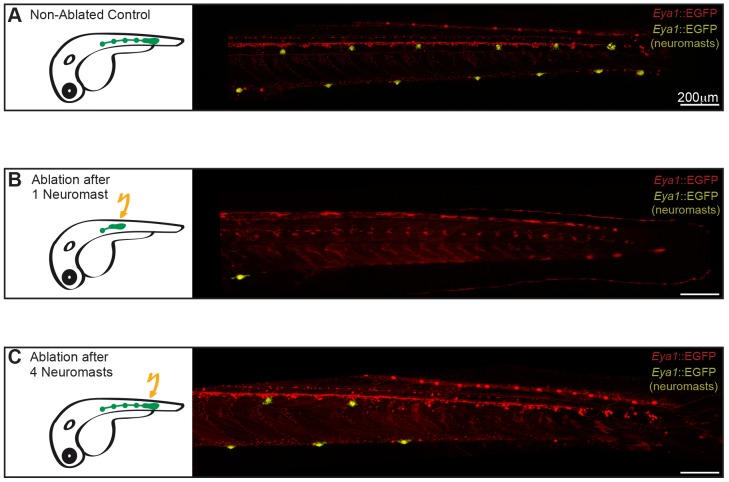
**A common primordium for ventral and midline pLL neuromasts.** (A,B) Neuromasts of the two parallel pLLs observed in wild-type larvae (A) are lost when the primordium is ablated early on (B) (*n*=4). (C) When the primordium is ablated after the deposition of three or four neuromasts, both midline and ventral pLL are present anterior but not posterior to the ablation site (*n*=4). Images in A-C were stitched and pseudocoloured to highlight neuromasts. Total expression pattern of Tg(*Eya1*:EGFP) is depicted in red, and EGFP^+^ neuromasts are shown in yellow.

Based on abundant data from zebrafish studies in which multiple migrating primordia have been shown to generate different pLLs (Ghysen and Dambly-Chaudière, 2007; Ledent, 2002; Nuñez et al., 2009; Sapède et al., 2002), it was largely assumed that each pLL in medaka derived from an independent primordium during embryonic development (Sapède et al., 2002). We used Tg(*Eya1*:EGFP) to follow embryonic pLL morphogenesis dynamically, and observed a primordium that delaminates near the otic vesicle from the lateral line placode at stage 26 and migrates posteriorly (Fig. 2A-B) along the myoseptum to reach the tail about three days later (Fig. 2C). Interestingly, this primordium generates approximately ten neuromasts that are deposited along the mindline – the number of neuromasts later found at the ventral pLL (Fig. 2C). Iterative observation of individual Tg(*Eya1*:EGFP) embryos indicated that the neuromasts produced by the primordium migrate ventrally and form the ventral pLL (Fig. 2D-E′; Movie 1). Intriguingly, we could never detect a second primordium as reported in zebrafish and, yet, we could observe the appearance of about seven midline pLL neuromasts distributed along the myoseptum on the following days (Fig. 3A; Fig. 1C,D). These results suggest either the presence of a second *eya1-*negative primordium, or different mechanisms for generating neuromasts in the midline and in the ventral pLLs.

To investigate the origin of the midline pLL neuromasts, we used two-photon laser ablations to remove the primordium just after the first neuromast was deposited (Fig. 3B, scheme). Ablation of primI in early zebrafish results in the absence of the primI-produced neuromasts, but it does not affect the migration of primII nor the generation of primII-derived neuromasts (López-Schier et al., 2004). After ablation, Tg(*Eya1*:EGFP) embryos were developed until juvenile stages. We observed that although the control side displayed complete ventral and midline pLLs, early primordium ablation on the experimental side resulted in the absence of all neuromasts both on the ventral and the midline pLLs (*n*=4 juveniles analysed; control side: 9.5±1.3 ventral neuromasts and 5.5±1 midline neuromasts; experimental side: 1 ventral neuromast and no midline neuromasts) (Fig. 3A,B). Later primordium removal resulted in the absence of neuromasts posterior to the ablation site in the ventral and the midline pLL (*n*=4) (Fig. 3C, scheme) but no evident phenotype anterior to the ablation site (Fig. 3C). These results strongly suggest that a single primordium is responsible for the generation of the two parallel pLLs in medaka, ruling out the one-primordium one-lateral-line concept.

**Fig. 4.**
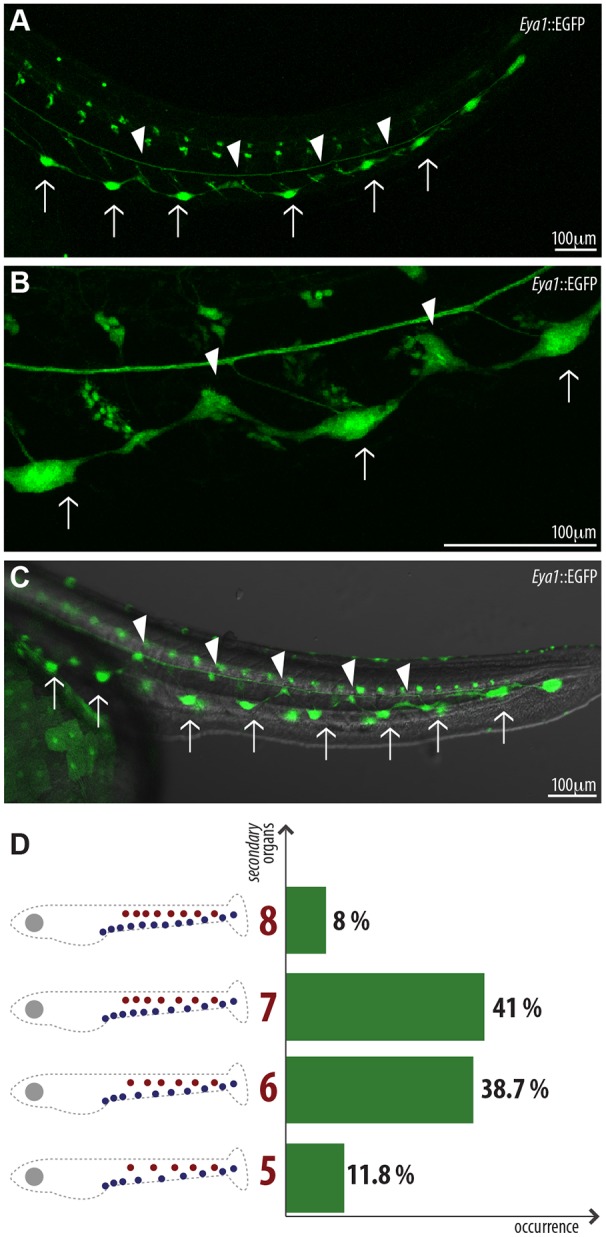
**mpLL neuromasts are formed between consecutive vpLL neuromasts.** (A) Stage 32 Tg(*Eya1*::EGFP) embryo. After ventral migration of primary organs, smaller neuromasts (arrowheads) appear between pairs of vpLL neuromasts (arrows) in an anterior-posterior manner. (B,C) These new organs, secondary neuromasts, start migrating dorsally (B) towards the horizontal myoseptum, where they will constitute the mpLL (C). (D) Distribution of secondary neuromast numbers in the population of Tg(*Eya1*::EGFP) (*n*=212 pLLs). We also observed one pLL displaying nine secondary organs (not shown).

If the two parallel pLLs come from the same primordium, when do they split apart? And when do they reach their final destination? By following a 4D approach on Tg(*Eya1*:EGFP), we observed that ventral migration of the neuromasts occurs soon after the primordium deposits each organ (Fig. 2D-D″; Movie 1). Therefore, the most anterior neuromasts start their ventral migration while the primordium is still on its way towards the tail (Fig. 2D-E′; Movie 1). After the initiation of ventral migration, we detected the appearance of new neuromasts (hereafter termed secondary neuromasts) between the ones that were originally deposited by the primordium (hereafter termed primary neuromasts) (Fig. 4A,B). The temporal appearance of secondary neuromasts follows the same anterior-posterior dynamics observed in the primary neuromasts. Secondary neuromasts are formed between all pairs of consecutive primary neuromasts (Fig. 4A,C), excluding the first two and last pairs. This alternating pattern resembles our previous observations comparing the ventral and the midline pLLs and suggests that secondary neuromasts are forming the midline pLL. Indeed, secondary neuromasts start migrating dorsally soon after being formed (Fig. 4B; Movie 2), and reach their final position just dorsal to the myoseptum (Fig. 4C). Overall, our results show that the two pLLs in medaka are formed in different manners and involve opposite migration. The ventral pLL is formed by neuromasts that are deposited in the midline by the primordium and then migrate ventrally. The midline pLL is formed by neuromasts that originate at the ventral part of the fish and migrate dorsally to the myoseptum (Fig. 4C).

### Individual cell migration initiates secondary neuromast formation

The formation of secondary neuromasts could be driven by signals outside or within the pLL system. Empty inter-somitic spaces could function as a call for the generation of secondary organs (Nuñez et al., 2009), but our data show that one secondary neuromast will always form between neighbour primary neuromasts regardless of their distance (three to five somites apart). Interestingly, the system displays natural variation in the number of primary neuromasts while maintaining the same distribution of organs. The number of mpLL neuromasts in Tg(*Eya*1:EGFP) ranges from five to eight (Fig. 4D). Larvae with five mpLL neuromasts display secondary organs between a pair of primary organs (100%; *n*=70 secondary neuromasts, *n*=14 pLLs) and so do larvae displaying eight secondary neuromasts (100%; *n*=48 secondary neuromasts, *n*=6 pLLs). This observation suggests that the number of secondary neuromasts is not decided by the empty spaces in the surrounding tissues (the occupancy theory), but rather is an intrinsic property of the lateral line systems.

**Fig. 5.**
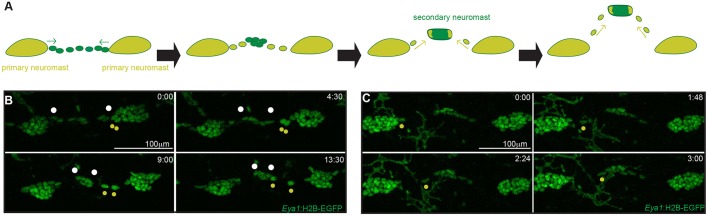
**Secondary neuromasts are formed by individual migration of ICs and cells from primary organs.** (A) Schematic of secondary neuromast formation. (B) Stills from Movie 3, stage 30-32 Tg(*Eya1*:H2B-EGFP) showing the coalescence of ICs (white dots) and migration of cells from primary neuromasts (yellow dots) to form a secondary organ. The white dots indicate the most distant ICs – the closer the dots, the more grouped the cells. (C) Stills from a time-lapse movie of stage 30-32 Tg(*Eya1*:H2B-EGFP) showing that even after the secondary organs started dorsal migration, *eya1^+^* cells continue to leave primary neuromasts and incorporate into secondary neuromasts.

Our previous observations demonstrated that the mpLL neuromasts are formed after vpLL neuromasts, which temporally resembles the formation of new neuromasts from ICs in zebrafish (Ghysen and Dambly-Chaudière, 2007; Grant et al., 2005; López-Schier and Hudspeth, 2005; Nuñez et al., 2009; Whitfield, 2005). There, cells that were deposited by the primordium between consecutive neuromasts re-enter the cell cycle in a process that involves Erbb4 signalling and glial cells (Grant et al., 2005; López-Schier and Hudspeth, 2005; Lush and Piotrowski, 2014; Sánchez et al., 2016). We followed secondary neuromast formation in 4D and could not detect proliferating ICs (*n*=14 secondary neuromast formation in 11 embryos). Intriguingly, instead we observed that all six to eight ICs group together in the middle of two consecutive primary neuromasts (Fig. 5A,B; Movie 3), start dorsal migration (Movies 2 and 4) and later form a secondary neuromast. In addition, *eya1^+^* cells escape from primary neuromasts and get incorporated in the forming secondary organ (Fig. 5A,B, yellow dots), a process that continues during dorsal migration (Fig. 5B,C; Movies 2 and 4). We observed migration of cells from primary neuromasts in all cases of secondary neuromast formation (*n*=34 movies, *n*=26 embryos) and using different transgenic lines [Tg(*Eya*1:EGFP), Tg(*Eya*1:H2B-EGFP) and Gaudi*^LoxPOUT^*; Movies 2 and 4]. Our movies also indicate that primary neuromasts contribute cells that join secondary-forming organs from both sides. Altogether, our data shows that secondary neuromasts in medaka are formed by two types of individually migrating cells, i.e. *eya1^+^* ICs and *eya1^+^* cells from primary neuromasts.

### Cxcr4b is necessary for secondary organ formation

**Fig. 6.**
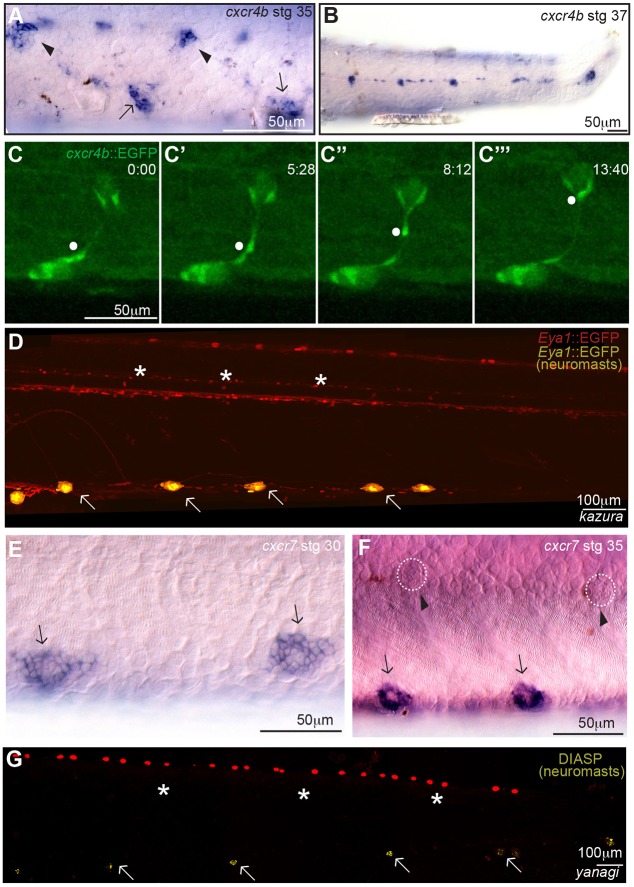
**CxcRs are expressed during and are necessary for secondary organ formation.** (A,B) *In situ* hybridisation using a *cxcr4b* antisense probe indicates expression of the chemokine receptor in primary neuromasts (arrows in A), forming secondaries (arrowheads in A) and ICs (*n*=2, stage 35). Expression of *cxcr4b* is detected only in secondary organs at late embryonic stages (B) (*n*=3, stage 37). (C-C‴) Time lapse of Tg(*cxcr4b*:EGFP) showing an EGFP^+^ cell migrating from a ventral to a midline neuromasts (white dot). (D) *kazura* mutants that display primary organs (arrows) do not generate secondary organs (asterisks). (E,F) *cxcr7* is expressed in the deposited organs (arrows in E). Expression is still detectable after ventral migration (arrows in F), but not in ICs nor in secondary organs (arrowheads and dotted circles in F). (G) *yanagi* mutants that display primary organs (arrows) do not generate secondary organs (asterisks). Neuromasts were detected either with DiAsp staining (G) or using Tg(*Eya1*:EGFP) embryos (D). Images in D and G were stitched and psuedocoloured to highlight neuromasts. Pigmented background (G) or total expression pattern of Tg(*Eya1*:EGFP) (D) are depicted in red, and DiAsp^+^ (G) or EGFP^+^ (D) neuromasts are shown in yellow.

Secondary neuromasts are generated by individual migration of ICs and cells from primary neuromasts. The Cxcl12a/Cxcr4b/Cxcr7b pathway is crucial for the collective migration of the primordium (David et al., 2002; Haas and Gilmour, 2006; Yasuoka et al., 2004; Valentin et al., 2007) and for individual cell migration of primordial germ cells (PGCs) in medaka and zebrafish (Boldajipour et al., 2008; Doitsidou et al., 2002; Herpin et al., 2008; Knaut et al., 2003; Sasado et al., 2008). Expression of *cxcr4b* and *cxcl12a* in medaka (Herpin et al., 2008; Yasuoka et al., 2004) largely matches that reported for zebrafish during primordium migration, with *cxcr4b* expressed in the primordium and in the deposited primary neuromasts, and *cxcl12a* distributed along the horizontal myoseptum (Fig. S2). At later stages, however, we noticed *cxcr4b* expression in the ICs (Fig. S2), in the migrating cells that form the secondary neuromasts and in the secondary organs (Fig. 6A,B). We complemented this with 4D imaging of Tg(*cxcr4b*:EGFP) embryos (Fig. S2) to show EGFP^+^ cells that migrate from primary to secondary organs (Fig. 6C-C‴; Movie 5). By the time secondary neuromasts reached their final position, *cxcr4b* expression was restricted to secondary neuromasts and absent in primary ones (Fig. 6B). The ligand, however, could not be detected in secondary organs (Fig. S2). The dynamic expression of *cxcr4b* suggests that the same chemokine receptor involved in collective migration of the primordium might play a later role during secondary organ formation.

To functionally test the role of Cxcr4b in secondary neuromast formation, we made use of the *cxcr4b* mutant *kazura*, which exhibits severe phenotypes in the pLL due to the impaired migration of the primordium (Yasuoka et al., 2004). Although most maternal/zygotic (m/z) *kazura* mutants do not display pLL neuromasts (59.2%; *n*=142 pLLs), a proportion manages to generate up to five neuromasts (36.6%; *n*=142 pLLs) (Yasuoka et al., 2004). In the few cases in which *kazura* mutants display six or seven neuromasts (4.2%; *n*=142 pLLs), all were primary organs (Fig. 6D). We only observed neuromasts along the vpLL, meaning they had reached their final position but could not form secondary neuromasts. Our results indicate a role for Cxcr4b in secondary neuromast formation because primary neuromasts in *kazura* mutants fail to produce secondary organs.

### Cxcr7 is necessary for secondary organ formation

Next, we carried out *in situ* hybridisations for *cxcr7*, a second receptor for Cxcl12a that in zebrafish is necessary for generating a signalling gradient along the primordium by acting as a sink for Cxcl12a at the rear (Dambly-Chaudière et al., 2007; Donà et al., 2013; Valentin et al., 2007). We detected a clear signal at the rear of the primordium as well as in deposited primary organs (Fig. S2). At later stages, however, *cxcr7* is not observed either in the ICs or in forming secondary organs (Fig. 6E,F). The viable mutant *yanagi* (Sasado et al., 2008; Yasuoka et al., 2004) allows exploration of the function of Cxcr7 during secondary organ formation. Similar to *kazura* mutants, m/z *yanagi* mutants displayed a strong phenotype with most pLLs lacking all neuromasts (56.7%; *n*=60 pLLs) although a fifth of the mutant larvae still display eight or more primary neuromasts (20%; *n*=60). Among these, however, most larvae showed no secondary neuromasts (66%; *n*=12 pLLs) (Fig. 6G) and the remaining just one secondary neuromast (33%; *n*=12 pLLs). Our results indicate that primary neuromasts in *yanagi* mutants fail to produce secondary organs, and therefore indicate a role for Cxcr7 in secondary neuromast formation.

### Wild-type cells rescue secondary organ formation in *kazura* and *yanagi* mutants

To understand how secondary organs fail to form in *kazura* and *yanagi* mutants, we followed the dynamics of ICs in both genotypes. Live imaging of Tg(*Eya1*:EGFP) *kazura* mutants revealed that most ICs, instead of grouping together in between organs, migrate back to the closest primary organ (Movie 6) (*n*=8). Secondary organs therefore fail to form in *kazura* mutants because there are insufficient ICs in place (Movie 7). *yanagi* mutant ICs, however, are seen dynamically shuttling between primary neuromasts, but are capable of neither coalescing nor being picked up by those organs (Movies 8 and 9) (*n*=5). Our 4D data on the mutants strongly implies that Cxcr4b and Cxcr7 have distinct roles during secondary organ formation.

**Fig. 7.**
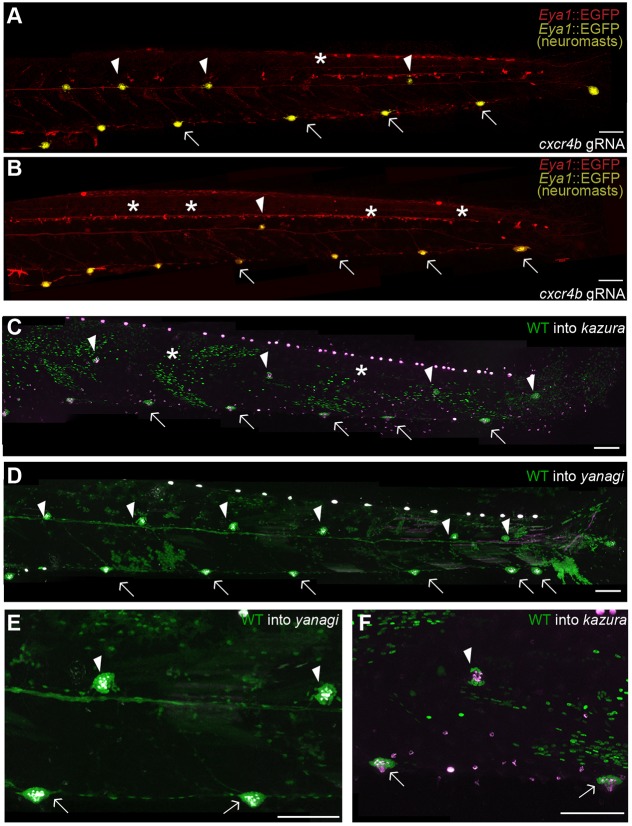
**Wild-type cells rescue secondary organ formation in *kazura* and *yanagi* mutants.** (A,B) Injection of gRNAs against *cxcr4b* results in Tg(*Eya1*:EGFP) larvae lacking some (A, asterisk) or many (B, asterisks) secondary neuromasts. (C-F) When wild-type EGFP^+^ cells are transplanted to *kazura* (C,F) or *yanagi* (D,E) mutants, they can rescue secondary organs (arrowheads in C-F) that contain functional hair cells as seen by DiAsp staining (magenta). All rescued secondary organs contain EGFP^+^ cells (E,F). Images were treated as in Fig. 6. Arrows indicate primary organs, arrowheads secondary organs and asterisks missing secondary organs.

In zebrafish wild-type*/cxcr4b* chimeric primordia, wild-type cells are maintained at the front of the primordium whereas mutant cells located in the middle and rear part eventually form mutant organs (Haas and Gilmour, 2006). In order to complement our observations in the *kazura* mutant, we exploited this internal organisation of a chimeric primordium by injecting two guide-RNAs (gRNAs) directed against *cxcr4b*. Both gRNAs worked *in vitro* and *in vivo* on the *cxcr4*b locus (Fig. S3) and similar approaches have been successfully used in medaka to generate F0 chimeras (Stemmer et al., 2015b). We observed that all injected embryos displayed pLLs on both sides when analysed at the larval stage. A proportion of these, however, displayed an aberrant ratio of primary to secondary organs (21.7%; *n*=198) (Fig. 7A,B), with extreme cases in which secondary organs were almost entirely absent despite a completely formed ventral pLL (Fig. 7B). We genotyped the embryos missing at least one secondary organ and detected a truncated allele of *cxcr4b* in 100% of the cases (7/7 embryos), therefore linking the aberrant phenotype to the mosaic genotype.

To explore secondary organ formation further, we generated chimeric embryos in which wild-type EGFP^+^ cells were transplanted into either *kazura* or *yanagi* mutants. We observed that the transplanted wild-type cells rescue the full migration of the primordium in both mutants (*n*=5 larvae each). Furthermore, wild-type cells also rescued the generation of mature secondary organs in *kazura* and *yanagi* backgrounds (Fig. 7C-F). Rescues only happened when the posterior lateral line contained EGFP^+^ cells, favouring a self-organising property of the pLL in generating secondary organs. Interestingly, transplantations revealed that every single secondary organ generated in *kazura* mutants (*n*=5) and all but one secondary organ generated in *yanagi* mutants (92.3%; *n*=13) contained wild-type EGFP^+^ cells. Altogether, our results show that Cxcr4b and Cxcr7 are used iteratively during posterior lateral line formation in medaka, initially driving the collective migration of the primordium and later in secondary neuromast formation.

## DISCUSSION

Remarkable plasticity in body shape has made teleost fish a very diverse animal class, containing roughly 80% of all vertebrate species. This plasticity comes with inherent problems, i.e. the changing ratio among organ sizes, different proportions of cell types within and expanded regions that need to be innervated and vascularised. We dynamically followed the posterior lateral line formation in an uncharacterised species to reveal a counter-intuitive morphogenetic event, in which a ventral pLL is generated along the myoseptum and migrates ventrally, whereas the midline pLL is generated at ventral positions and migrates dorsally.

### Internal organisation of the lateral line system in medaka

We found a variable number of neuromasts in the pLL among medaka larvae. It is unlikely that this reflects polymorphisms in the population, as most larvae display different numbers of neuromasts in the left and right pLLs (73%; *n*=90 pairs of pLLs). The same genetic information can therefore result in different morphological outputs on each side of the same organism. How is the relative number of midline and ventral pLL neuromasts defined? If the two lines compete for empty spaces along the medaka trunk, we would expect that a fish with a high number of primaries would generate more secondaries, and one with fewer primaries would need several secondaries to occupy all available positions. However, a pair of primary organs generates just one secondary neuromast, regardless of the total number deposited by the primordium. Taken together, our data indicate that the decision to generate a secondary organ resides within the lateral system and is not triggered by external cues. The autonomous control of the lateral line organs was highlighted by Ghysen and Dambly-Chaudière in the context of neuromast size (Ghysen and Dambly-Chaudière, 2007), and our results extend that concept to the initial formation of the system. We believe that this mode of lateral line morphogenesis ensures a stereotypic distribution and ratio of mature organs.

### Same cells, same molecules, different morphogenetic events

There are three known ways of generating a fish neuromast during homeostatic conditions: (1) direct deposition by the primordium during early embryogenesis (Ghysen and Dambly-Chaudière, 2007; Haas and Gilmour, 2006; Lecaudey et al., 2008; Nechiporuk and Raible, 2008), (2) proliferation of ICs between consecutive neuromasts during the early larval stages (Ghysen and Dambly-Chaudière, 2007; Grant et al., 2005; López-Schier and Hudspeth, 2005; Nuñez et al., 2009; Whitfield, 2005) and (3) neuromast fission or stitching, during late larval and young adult stages (Ghysen and Dambly-Chaudière, 2007; Ledent, 2002; Sapède et al., 2002; Wada et al., 2010). We show that midline pLL neuromasts in medaka are formed by the coalescence of ICs and cell recruitment from primary organs. The proportion of ICs and cells coming from the primary organs varies along the anterior-posterior axis, but includes in all cases the total amount of ICs between primary organs (six to eight Eya1^+^ cells). Interestingly, this process does not involve cell proliferation, which resumes only when secondary organs reach their final destination (A.S. and L.C., unpublished). We report a novel way to make embryonic neuromasts that results in a new, separate lateral line. Our data using *kazura* and *yanagi* mutants indicate that these receptors are being re-utilised by the system to generate more neuromasts, although mediating different processes, i.e. individual cell migration. Overall, *eya1*-expressing cells use the same chemokine receptors to generate identical neuromasts in two different manners.

### Distinct roles for Cxcr4b and Cxcr7 during secondary organ formation

The generation of chimeras wild type*/kazura* and wild type*/yanagi* revealed that wild-type cells can rescue both primordium migration and secondary organ formation. The distribution of wild-type EGFP^+^ cells along the pLL of each mutant suggests similar roles of Cxcr4b and Cxcr7 during primordium migration to those reported in zebrafish. In wild-type*/kazura* chimeras, H2B-EGFP^+^ cells accumulate towards the most posterior neuromasts (as expected if Cxcr4b is necessary at the leading part of the primordium) whereas in wild-type*/yanagi* chimeras EGFP^+^ cells accumulate towards the most anterior neuromasts (as expected if Cxcr7 is necessary at the rear of the primordium). The proportion of EGFP^+^ cells that rescue the mutant phenotype was different in each case. To rescue primordium migration fully in *yanagi* mutants, the lateral line should contain a majority of wild-type EGFP^+^ cells; if there are just a few wild-type cells in the initial primordium, these will be deposited on the first neuromasts and the primordium will stop caudal migration (observed in two larvae). On the other hand, in *kazura* mutants, a few EGFP^+^ cells were enough to rescue full primordium migration.

Although we cannot exclude the possibility that putative early defects in primordium migration might contribute to the phenotypes that we report for *kazura* and *yanagi* mutants, our results suggest that both Cxcr4b and Cxcr7 are used during secondary organ formation. The high ratio of transplanted cells prevents us from making statements about the specific role of Cxcr7 during secondary organ formation. Integrating the expression data, it might well be that Cxcr7 is operating from the primary organs in an indirect manner. On the other hand, the fact that every secondary organ contains EGFP^+^ cells indicates that *cxcr4b* is necessary in the migrating cells initiating the formation of new neuromasts, in accordance with the expression of *cxcr4b* in ICs. *cxcr4b*^+^ ICs could be responding to a Cxcl12a ligand. Based on our data, we cannot state whether *cxcr4b^+^* ICs are migrating in a Cxcl12a-dependent or -independent manner. Interestingly, it has been shown that fibroblast growth factor (FGF) works as an attractant cue for isolated cells towards the primordium during early migration in zebrafish (Breau et al., 2012), and functions as an antagonist for Cxcr4b signalling. Our data suggests that this same interaction could modulate secondary organ formation in medaka, as *cxcr4b* mutant cells were observed shuttling back to the primary organs instead of coalescing between them. Additional experiments will be needed to address whether a fine-tuning of the opposing roles of FGF and Cxcr4b triggers secondary organ formation in medaka.

### On the diversity of pLL patterns

The embryonic development of the pLL has been extensively studied in zebrafish, *Astyanax mexicanus* and *Thunnus thynnus* (Ghysen et al., 2012, 2010; Pichon and Ghysen, 2004; Sapède et al., 2002), all of which display two pLL primordia during embryonic development and a species-specific pattern of neuromasts at adult stages. This has led to the notion that the plethora of different patterns of pLLs arises primarily during post-embryonic stages of teleost fish, building on largely identical embryonic patterns (Nuñez et al., 2009; Pichon and Ghysen, 2004). Our results complement this assumption by showing pronounced changes during embryonic organogenesis of lateral line patterns and place medaka as the first reported teleost species to have only one pLL primordium building two parallel sensory lines during embryogenesis. This distribution of neuromasts resembles the complete pattern generated by primII in zebrafish (Nuñez et al., 2009). Interestingly, the neuromasts located along the horizontal myoseptum in an adult zebrafish (Ghysen and Dambly-Chaudière, 2007) are lost when primII is ablated during embryogenesis (Nuñez et al., 2009). We believe that this primordium might constitute a good example of heterochrony between the more basal zebrafish and the neoteleost medaka (Wittbrodt et al., 2002).

Overall, our results show that one primordium generates two different lateral lines in medaka, whereas two primordia are present in zebrafish, *Astyanax* and tuna. This raises two interesting questions. First, when did medaka lose a primordium during evolution? This can be addressed dynamically by following pLL development in close relatives of medaka such as platyfish and stickleback and we believe the Tg(*Eya1*:EGFP) constitutes an ideal tool. Second, how can a species lose an entire developmental module, i.e. a primordium? Recent work has demonstrated that small changes, either in non-coding sequences or in temporal expression of key regulators, can have drastic effects on the unfolding of morphogenetic processes (Indjeian et al., 2016; O'Brown et al., 2015; Urbansky et al., 2016). The availability of genomic data in different fish species that contain either one or two primordia allows for a comparative analysis, which could yield interesting and novel insights into the evolution of lateral lines, and more generally the evolution of morphogenetic processes.

## MATERIALS AND METHODS

### Fish stocks

Medaka (*Oryzias latipes*) stocks were maintained as previously described (Koster et al., 1997). Fish were kept as closed stocks in a fish facility built according to the local animal welfare standards (Tierschutzgesetz §11, Abs. 1, Nr. 1), and animal experiments were performed in accordance with European Union animal welfare guidelines. The facility is under the supervision of the local representative of the animal welfare agency. Fish were maintained in a constant recirculating system at 28°C on a 14 h light/10 h dark cycle (Tierschutzgesetz 111, Abs. 1, Nr. 1, Haltungserlaubnis AZ35–9185.64 and AZ35–9185.64/BH KIT).

The strains used in this study are: Cab (wild-type population), *kazura* (*cxcr4b* mutant) and *yanagi* (*cxcr7* mutant) (Sasado et al., 2008; Yasuoka et al., 2004), *Gaudi*^LoxPOUT^ (ubiquitous H2B-EGFP expression) (Centanin et al., 2014). The following transgenic lines were generated by I-*Sce*I mediated insertion, as previously described (Rembold et al., 2006a; Thermes et al., 2002): Tg(*Eya1*:EGFP), Tg(*Eya1*:mECFP), Tg(*Eya1*:H2B-EGFP) and Tg(*cxcr4b*:EGFP). For generation of Tg(*Eya1*:EGFP), a 3.2 kb fragment of the medaka *eya1* promoter sequence was retrieved from the fosmid GOLWFno481_e16 (NBRP Medaka) by PCR with a specific primer set (forward: ATCCCTGCAGCCCCACATGA; reverse: AGCGAGCTCCCTCACAAGCC). The PCR fragment was A-tailed and cloned into pGEM T-easy (Promega), and subcloned using *Apa*I/*Sal*I sites into I-*Sce*I vectors (Rembold et al., 2006b; Thermes et al., 2002) already containing EGFP, mECFP or H2B-EGFP. For generation of Tg(*cxcr4b*:EGFP), a 1.9 kb fragment upstream of the presumptive first exon of the endogenous *cxcr4b* locus was amplified from Cab genomic DNA using a specific primer set (forward: TTTAGAAGCAGCGGTGTGC; reverse: CAGTATAAAAGGCGCGTGCGAG). The PCR fragment was cloned into an I-*Sce*I vector (Rembold et al., 2006b; Thermes et al., 2002) already containing EGFP.

### Cxcr4b gRNAs

Two gRNAs targeting the coding sequence of the endogenous *cxcr4b* locus in Medaka were designed *in silico* using CCTop (Stemmer et al., 2015b) and gRNAs were synthesised as previously described (Stemmer et al., 2015b). Briefly, oligo annealing (sgRNA-1_F: TAGGCGAGGGCGATGCCACCTA; sgRNA-1_R: AAACTAGGTGGCATCGCCCTCG; sgRNA-2_F: TAGGTCAGGGTCAGCAGTCCAT; sgRNA-2_R: AAACATGGACTGCTGACCCTGA) was used to generate specific gRNAs which were then ligated into an *Eco*31I (Thermo Fisher Scientific) linearised DR274 plasmid (Addgene clone number: #42250). This was followed by *in vitro* RNA transcription using T7 MEGAshortscript Kit (Ambion) and a clean-up step using RNAeasy kit (Qiagen). The gRNAs were co-injected at 10 ng/μl each along with 150 ng/μl of CAS9 mRNA into Tg(*Eya1*:EGFP) one-cell-stage medaka embryos. The sequences of *cxcr4b* gRNAs are GUGAAAACCUGGUACUUCGGAGG and CAAGUGGAUUUCUAUCACCGAGG.

### Live-imaging sample preparation

Dechorionated embryos or hatchlings were anaesthetised using Tricaine (Sigma-Aldrich, A5040-25G). Tricaine (2 g) was dissolved in 500 ml of ddH_2_O together with 5 g of NaH_2_P_4_.2H_2_O (Grüssing, 12133) and used as a 20× stock solution. Embryos were mounted in 0.6% low melting agarose in ERM (embryo rearing medium) (Rembold et al., 2006a) on glass-bottomed dishes (MatTek corporation). They were covered with 1× Tricaine in 1× ERM to prevent dehydration. After imaging, dechorionated embryos were removed out of agarose and allowed to recover in ERM.

### Whole-mount *in situ* hybridisation

Probe generation and *in situ* hybridisations were performed as previously reported (Stemmer et al., 2015a). Briefly, partial *eya1*, *cxcr4b*, *cxcr7* and *cxcl12a* cDNA clones were linearised to generate an anti-sense and control sense probes. The fragments were purified with InnuprepPCRpure (Analytic Jena), and used in reactions with T7, SP6 (antisense) or T3 (sense) RNA polymerases and Dig-UTP, to obtain labelled probes that were stored at −20°C in hybridisation solution. Hybridisations were performed overnight at 65°C and samples were incubated afterwards with an antibody against anti-digoxigenin conjugated with AP Fab fragments (1:2000; Roche, 11093274910). Staining was performed using NBT/BCIP (Roche) and followed under a binocular microscope.

### DiAsp/DAPI staining

Hair cells in living embryos were detected by using the vital dye 4-Di-2-ASP (Sigma-Aldrich) as previously described (Collazo et al., 1994; Sapède et al., 2002). Briefly, live samples were incubated for 5-10 min in a 5 mM DiAsp solution, washed three times in ERM and observed under a fluorescent binocular. Alternatively, samples were treated with DAPI (5 mg/l) for 5 min and washed three times with ERM before mounting them for confocal microscopy.

### Imaging and image analysis

Embryos were screened and imaged using an Olympus MVX10 binocular coupled to a Leica DFC500 camera, a Macro-scope Nikon AZ100 coupled to a Nikon C1 confocal, and the confocal laser-scanning microscopes Leica TCS SPE (40× oil objective) and Leica TCS SP5 II (10× dry, 40× dH2O objectives). For long-term time-lapse imaging, we used a Microscope Slide Temperature Controller (Biotronix). *In situ* hybridisation pictures were taken using an Axio microscope connected to an HRC camera (Zeiss).

All image analysis was carried out using standard Fiji software. Image stitching was performed automatically using 2D and 3D stitching plug-ins on ImageJ or using Photoshop to align images manually. Pseudocolouring of pigments and/or *eya1*^+^ spinal cord neurons was achieved using Fiji software.

### Two-photon laser ablation

The lateral line primordium of Tg(*Eya1*:GFPs) medaka embryos was ablated either at 2 (early) or at 3 (middle-way) days post-fertilisation (dpf). A TriM Scope 2-photon microscope (LaVision BioTec, Bielefeld, Germany) mounted on a Nikon FN-1 upright stand, equipped with a Chameleon Ultra II femtosecond Ti:Sa laser (Coherent, Dieburg, Germany) and a water-dipping 16× NA 0.8 LWD objective (Nikon) was used. Ablations were performed with 740 nm wavelength and laser power between 150 and 700 mW at the objective output, depending on the depth of the ablation.
